# Knowledge on birth preparedness and complication readiness among expecting couples in rural Tanzania: Differences by sex cross-sectional study

**DOI:** 10.1371/journal.pone.0209070

**Published:** 2018-12-28

**Authors:** Fabiola V. Moshi, Alex Ernest, Flora Fabian, Stephen M. Kibusi

**Affiliations:** 1 School of Nursing and Public Health, the University of Dodoma, Dodoma, Tanzania; 2 School of Medicine and Dentistry, the University of Dodoma, Dodoma Tanzania; University of Michigan Medical School, UNITED STATES

## Abstract

**Background:**

Inadequate knowledge of birth preparedness and complication readiness (BPCR) among expecting couples delays timely access to maternal emergency services. The aim of this study was to assess knowledge on birth preparedness and complication readiness and how men and women differ" among expecting couples in a rural setting of Rukwa Region, Tanzania.

**Methods:**

A community-based cross-sectional study targeting pregnant women and their partners was performed from June 2017 to October 2017. A total of 546 couples were sampled using three-stage probability sampling techniques and then interviewed using a structured questionnaire. The mean score difference was sought using independent t-test. Multiple linear regressions were performed to determine the predictors of knowledge.

**Results:**

There was a significant difference in mean knowledge scores between pregnant women (M = 5.58, SD = 4.591) and male partners (M = 4.37, SD = 4.285); t (1085) = -4.525; p<0.001. Among women, BPCR levels were positively influenced by age (β = 0.236; p<0.01), having ever heard about birth preparedness (β = 0.176;p<0.001), being of Mambwe ethnicity (β = 0.187; p<0.001), living near a health center rather than a dispensary (β = 0.101;p<0.05) and having had a prior preterm delivery (β = 0.086;p<0.05). Access to media through radio ownership negatively influenced BPCR levels among both women (β-.119; p<0.01) and men (β = -0.168; p<0.0001). Among men, the BPCR knowledge was only positively influenced by having ever heard about birth preparedness (β = 0.169;p<0.001), age at marriage (β = -0.103; p<0.05), and having completed either primary (β = 0.157;p<0.001) or secondary education (β = 0.131;p<0.01).

**Conclusion:**

Some important predictors of knowledge were revealed among women and men, but overall knowledge about birth preparedness and complication readiness was low. This study demonstrates inadequate knowledge and understanding at the community level about key elements of birth preparedness and complication readiness. In order to improve access to life-saving care for women and neonates, there is a pressing need for innovative community strategies to increase knowledge about birth preparedness and complication readiness. Such strategies are essential in order to reduce maternal and neonatal mortality in rural Tanzania.

## Introduction

Maternal death is a major public health problem globally. It is estimated that 216,000 maternal deaths occurred in 2015worldwide [1]. The major causes of these deaths were; maternal hemorrhage (44,200 deaths), complications of abortion (43,700 deaths), maternal hypertensive disorders (29,300 deaths), maternal sepsis and other maternal infections (23,800 deaths) and obstructed labor (18,800 deaths) [2]. Most of these deaths occurred in Sub-Saharan Africa (62%) and South Asia (24%) which altogether account for 86% of maternal mortality worldwide [3].

The risk of a woman dying due to maternal causes in developing countries is high: one woman in every 76 deliveries [4], while the risk in Tanzania is one in 44 deliveries [5]. Therefore, Tanzania ranks among the countries with one of the highest maternal mortality rates (MMR) worldwide [6]. The 2015 Tanzania Demographic Health Survey estimated Tanzania's MMR at 556/100,000 [7], which translates to six maternal deaths for every 1,000 live births in Tanzania.

Many different factors contribute to the existence of high maternal mortality and morbidity in the Global South, including countries such as Tanzania [8] and the reasons are complex and cross-cutting. At the community level, leading factors for high maternal mortality include knowledge gaps, fatalism (someone believes maternal health outcomes are determined in advance and cant be changed), low status of women and lack of emergency care access before, during and after delivery [9]. Pregnant women and their families often ignore early warning signs due to lack of adequate knowledge and information about danger signs during pregnancy and labor and therefore delay seeking care [9]. Women in the Global South rarely have meaningful decision-making power which impedes timely access to obstetric care[9]. Some women who have knowledge about pregnancy danger signs delay in seeking immediate care due to the submissive roles they have within the family[10].

Men in developing countries often have even less knowledge about reproductive health compared to their women partner[11]. Moreover, men in these countries often are controlling decision making around such timing and conditions of sexual relations, family size, and whether or not their partners utilize available health care services[12] which has also has a significant negative impact on maternal and neonatal outcomes.

Empowering men with necessary information about emergency obstetric conditions, and engaging them in birth preparedness and complication readiness (BPCR) is a vital strategy towards improving maternal services utilization[13]. One approach employed in the Global North to reduce maternal and neonatal mortality has been to include expecting fathers in the maternal and newborn health care system. The most common approach in the global north, among others, is to include men in regular prenatal checkups as well as parent training[14]. The state of male involvement in some countries is as high as 80% in Denmark [14] and 90% in Sweden [3]. Among many other factors, male involvement in BPCR may be a protective factor for women in the global north, as evidenced by the low MMR in 0–10 maternal deaths per 100,000 live births in the global north [15].

In the global south, male partners’ presence and participation at prenatal care visits varies greatly from 96% in the Maldives[16] to only 12% in Tanzania[17], 18% in Burundi [16] and 32.1% in Nigeria[11]. In the global south, male partners have limited knowledge about complications related to childbirth. One study examining determinants of male partner involvement in promoting facility deliveries found that 40% of study participants saw childbirth as a natural phenomenon that does not require men's participation, and 48.2% of them said that they would be ridiculed by their peers and seen as being "ruled" by their wives if they were seen accompanying them to health facilities for delivery[13,18].

Low levels of knowledge about obstetric danger signs and what to prepare for childbirth do influences the total level of a family’s 'BPCR. Birth preparedness facilitates a reduction in all three phases of delays to access maternal services. These delays include: a delay in decision-making to seek healthcare, a delay in reaching a health facility and a delay in obtaining appropriate care upon reaching a health facility[19]. These three delays have been well documented in the literature to impact access to skilled birth attendants and therefore contribute to maternal and neonatal deaths.

In 2012 Rukwa Region was the region in Tanzania with the highest MMR of 860 deaths per 100,000 live births[20]. At that time, facility birth rates were also the lowest in the country at 30.4%. Due to changes in regional geographic boundaries and a focus on increasing facility delivery, homebirth rates have decreased significantly. As of 2015, 65% of women were reaching health facilities to deliver their babies [7]. In this rural setting, social-cultural barriers have been documented to affect place of delivery. These include gender norms for decision-making, perception of childbirth as a low-risk event among both men and women, and fatalistic attitudes. These cultural factors have historically hindered the uptake of health facility childbirth in this region[18]. Before this study, little was known in this setting about expecting couples’ knowledge about BPCR. The aim of this paper was therefore to assess the level of knowledge of BPCR among expectant couples and identify predictors of knowledge based on gender and other key factors.

## Materials and methods

### Study design and setting

A community-based cross-sectional study was conducted in Rukwa Region from June 2017 until October 2017, among expecting couples from 45 villages in Rukwa Region. This region is located in the Southern Highlands of Tanzania and has a population of 1,004,539 people; 487,311 males and 517,228 females. The forecast for 2014 was 1,076,087 persons with a growth rate of 3.5%[21]. The region has the lowest mean age at marriage where males marry at the age of 23.3 years and 19.9 years for females and fertility rate of 7.3[21].

### Sampling method and sample size

#### Sampling technique

Two districts (Sumbawanga Rural District and Kalambo District) were conveniently selected from the four districts within Rukwa Region. Three staged multi-stage cluster sampling technique was used to obtain study participants. During first stage random samplings, all Wards in each district (12 Wards of Sumbawanga Rural District and 17 Wards of Kalambo District) were listed and by the use of the lottery method of random sampling, five Wards from Sumbawanga District and ten from Kalambo District were selected. During second stage random sampling, all villages in the selected Wards were listed and another simple random sampling was used to select fifteen villages from Sumbawanga Rural District and thirty villages from Kalambo District. The third stage sampling was a systematic sampling used to obtain households with pregnant women of 24 weeks gestation or below who were living with a male partner. At each visited household, a female partner was interviewed for the signs and symptoms of pregnancy or current known pregnancy, and any woman with amenorrhea for a minimum of two months was offered a urine pregnancy test. Those who consented were then tested during the home visit. Those with positive tests and women who already knew they were pregnant were educated about the study and consented to participate. Those who gave verbal and written consent to participate, their partners were consulted given a verbal and written consent to participate; couples which consent were enrolled in the study. If a selected household had no eligible participants or refused to be tested or was tested and refused to participate in the study, the household was skipped, and the next household was visited.

#### Sample size calculation

The sample size for couples who were involved in the study was calculated using the following formula [22].

$$n=\frac{{\left\{z\alpha \sqrt{\left[\pi_{0}\left(1-\pi_{0}\right)\right]}+z\beta \sqrt{\left[\pi_{1}\left(1-\pi_{1}\right)\right]}\right\}}^{2}}{{\left(\pi_{1}-\pi_{0}\right)}^{2}}$$

Where:

n = minimum sample size

Zα = Standard normal deviation (1.96) at 95% confidence level for this study

Zβ = standard normal deviate (0.84) with a power of demonstrating a statistically significant difference before and after the intervention between the two groups at 90%

π₀ = Proportion at pre- intervention (Use of skilled delivery in Rukwa region 30.4%[23]

π₁ _=_ proportion after intervention (Proportion of families which would access skilled birth attendant 51%) [19]

$\frac{n={\left\{1.96\surd [0.301\left(1‑0.301\right]+0.84\surd [0.51\left(1‑0.51\right)]\right\}}^{2}}{{\left(0.6‑0.51\right)}^{2}}$

n = 162 couples + 10% = 180

Therefore, the required sample size in the intervention group = 180 couples

Intervention: control ratio = 1:2. Therefore sample size in the control group = 360 couples

The baseline data was reported on both groups, the intervention group and control group which make a total of 546 couples

### Data collection procedure

Data were collected using semi-structured questionnaire. A research assistant asked questions and documented the responses among all study participants. Four trained research assistants (two from each district) were recruited, trained and participated in data collection. Questionnaires on knowledge about birth preparedness and complication readiness were adopted and modified from monitoring BPCR tools for maternal and newborn health[24] in prototype safe motherhood population-based survey questionnaire- section three Several studies have adopted this tool[25,26]. After modification, it contained five sections namely; socio-demographic information, obstetric history, danger signs (during pregnancy, labor and childbirth, 42-days post delivery and neonatal danger signs), antenatal care and preparations for childbirth. On knowledge on danger signs; respondents were required to recall danger signs they knew in four areas; during pregnancy, labor and childbirth, 42 days post-delivery and neonatal danger signs. Knowledge about antenatal care was measured in two areas; knowing the appropriate time for first antenatal care booking and the number of recommended antenatal visits. Knowledge about childbirth preparation was measured through recalling the preparations to be made for childbirth. Knowledge about birth preparedness and complication readiness is the total knowledge of danger signs, antenatal care and preparations for childbirth. The modified tool was translated from English to Swahili (local, national language), pretested by face to face validation, modified and then utilized.

### Data processing and analysis

The data were checked for completeness and consistencies; then were coded and entered in to computer using statistical package IBM SPSS version 23. Descriptive statistics were used to generate frequency distribution and cross tabulation were used to describe the characteristic of the study participants. Measures of central tendency were established on the outcome variable (knowledge preparedness and complication readiness), and comparison between a mean knowledge score of pregnant women and their partners was done using independent t-test. Bivariate analysis was done to determine predictors of knowledge about birth preparedness and complication readiness. All variables with a p-value of 0.2 and below were entered simultaneously in multiple linear regression to determine predictors of knowledge after adjusting for confounders.

### Ethical consideration

The proposal was approved by Ethical Review Committee of the University of Dodoma in Dodoma, Tanzania. Furthermore, a letter of permission was obtained from the Rukwa Regional Administration. Both written and verbal consents were sought from study participants after explaining the study objectives and procedures and their right to refuse to participate in the study at any time they were assured.

## Results

### Socio-demographic characteristics of study respondents

A total of 546 couples were included in the baseline study, with a response rate of 100%. The 546 pregnant women were no more than 24 weeks pregnant at the time of enrollment. The mean age of pregnant women was 25.57 years (sd = 6.810), and the mean age of their partners was 30.65 years (sd = 7.726). The majority of the couples were married (390, 71.4%), monogamous (469, 85.9), live on less than 1 dollar per day (382,70.0%), and obtain basic obstetric care services from dispensaries (452, 82.8%). Two hundred ninety-nine (54.8%) of pregnant women and 353 (64.7%) of their partner had primary level of education (Table 1).

**Table 1:** Socio-demographic characteristics of study respondents (N = 1092).

Character	Female (n_1_, %)	Male (n_2_, %)	Total (n_1_+n_2_)	p-value
	(n_1_ = 546)	(n_2_ = 546)	1092	
**Age (years)**				0.000
Less than 20	167(30.6)	27 (4.9)	194(17.8)	
21 to 25	156(28.6)	143(26.2)	299(27.4)	
26 to 30	105(19.2)	146(26.7)	251(23.0)	
31 to 35	55(10.1)	87(15.9)	142(13.0)	
36 and above	63(11.5)	143(26.2)	206(18.9)	
**Age at Marriage (years)**				0.000
Less than 18	395(72.3)	71(13.0)	466(42.7)	
19 to 24	147(26.9)	353(64.7)	500(45.8)	
25 and above	4(0.7)	122(22.3)	126(11.5)	
**Ethnic group**				0.001
Fipa	322(59.0)	367(67.2)	689(63.1)	
Mambwe	120(22.0)	118(21.6)	238(21.8)	
Others	104(19.0)	61(11.2)	165(15.1)	
**Marital status**				0.893
Cohabit	156(28.6)	154(28.2)	310(28.4)	
Married	390(71.4)	392(71.8)	782(71.6)	
**Education level**				0.000
Non-formal	230(42.1)	155(28.4)	385(35.3)	
Primary School	299(54.8)	353(64.7)	652(59.7)	
Secondary school or Higher	17(3.1)	38(7.0)	55(5.0)	
**Income per day**				0.254
Less than 1 dollar	399(73.1)	382(70.0)	781(71.5)	
More than 1 dollar	147(26.9)	164(30.0)	311(28.5)	
**Have access to media**				0.001
Yes	253(46.3)	308(56.4)	561(51.4)	
No	293(53.7)	238(43.6)	531(48.6)	
**Own mobile phone**				0.000
Yes	69(12.6)	234(42.9)	303(27.7)	
No	477(87.4)	312(57.1)	789(72.3)	
**Health facility**				
Dispensary	452(82.8)	452(82.8)	904(82.8)	**1**
Health Centre	94(17.2)	94(17.2)	188(17.2)	
**Distance to health facility(Km)**				
Less than 1	258(47.3)	259(47.4)	517(47.3)	
1 to 5	233(42.7)	232(42.5)	465(42.6)	0.998
More than 5	55(10.1)	55(10.1)	110(10.1)	

### Obstetric characteristics of pregnant women

One hundred and twenty (22%) of pregnant women were pregnant for their first time, and 426 (78%) were pregnant at least the second time. Only 122 women (22%) were nulliparous, 320 (58.6%) had one to four deliveries, and 106 (19.4) had five or more deliveries. Twenty-nine women (5.3%) had a prior preterm delivery, and only 13 (2.4%) had ever had previous cesarean section (Table 2).

**Table 2:** Frequency distribution of obstetric history of the female respondents (N = 546).

Variable	Female (n_1_, %)
	(n = 546)
**Gestation Age (Weeks)**	
≤ 16	116(21.2)
17–24	430(78.8)
**Gravidity**	
Prime-gravid	120(22.0)
Multiparous	426(78)
**Parity**	
Null-parous	120(22.0)
Para 1–4	320(58.6)
Para 5+	106(19.4)
**History of pre-term delivery**	
Yes	29(5.3)
No	517(94.7)
**History of Caesarean section**	
Yes	13(2.4)
No	533(97.6)

### Awareness about birth preparedness and complication readiness

Study respondents were asked if they had ever heard about birth preparedness and complication readiness. It was found that 452 (82.8%) of pregnant women and 444 (81.3%) of their partner had ever heard something about birth preparedness and complication readiness. The majority of study participants referenced health facilities as a source of information, which was the case among 423 (77.5%) pregnant women and 395 (72.3%) of their male partners.

### Knowledge about danger signs of obstetric complications

The mean score of knowledge of danger signs was 3.17 ± 3.793 among pregnant women, where minimum score was 0 and maximum score was 25. Among male partners, the mean score was 2.31 ± 3.298 where the minimum was 0 and maximum was 14. More than half of both men and women could not recall any danger signs during pregnancy (321, 58.8%), childbirth (370, 67.8%), 42-days post delivery (418, 76.6%), and the neonatal period 381 (69.8%), respectively. Among pregnant women, 48.0% could not recall any danger signs during pregnancy (n = 262) and 57.7% could not recall any danger signs during childbirth (n = 315). Knowledge during the post-partum period was similarly low with 66.3% of women unable to recall any dangers signs during 42-days post delivery (n = 362) and 58.2% unable to name a single neonatal danger sign (n = 318). Very small numbers of men and women knew at least three danger signs for each key period.

Results for men knowing three dangers signs for each period were as follows: 59 (10.8%) during pregnancy, 27 (5%) during labor, 37 (6.8%) during the postpartum period and 48 (8.8%) for neonates. Among women: 81 (14.8%), 33 (6%), 44 (8%) and 57 (10.4%) of pregnant women managed to mention at least three danger signs during pregnancy, labor, 42-days post delivery and the neonatal danger signs (Table 3).

**Table 3:** Sex differences on recalling danger signs during pregnancy, childbirth, 42-days post delivery and neonatal period.

Variables	Females (n_1_, %) 546	Males (n_2_, %) 546	Total(n_1_+n_2_) 1092	*p-value*
** Pregnancy Danger Signs**				0.010
Unable to recall any	262(48.0)	321(58.8)	583(53.4)	
Recalled one	105(19.2)	96(17.6)	201(18.4)	
Recalled two	98(17.9)	70(12.8)	168(15.4)	
Recalled three and above	81(14.8)	59(10.8)	140(12.7)	
**Labor and Birth Danger Signs**				0.018
Unable to recall any	315(57.7)	370(67.8)	685(62.7)	
Recalled one	121(22.2)	88(16.1)	209(19.1)	
Recalled two	77(14.1)	61(11.2)	138(12.6)	
Recalled three and above	33(6)	27(5)	60(5.5)	
**42-days postpartum danger Signs**				0.000
Unable to recall any	362(66.3)	418(76.6)	780(71.4)	
Recalled one	94(17.2)	73(13.4)	167(15.3)	
Recalled two	46(8.4)	18(3.3)	64(5.9)	
Recalled three and above	44(8.0)	37(6.8)	81(7.6)	
**Neonatal Danger Signs**				0.002
Unable to recall any	318(58.2)	381(69.8)	699(64.0)	
Recalled one	104(19.0)	68(12.5)	172(15.8)	
Recalled two	67(12.3)	49(9.0)	116(10.6)	
Recalled three and above	57(10.4)	48(8.8)	87(9.7)	

Regarding obstetric danger signs during pregnancy, vaginal bleeding was the most recalled obstetric danger sign by both pregnant women and their partners. This was cited by 192 (35.2%) of pregnant women and 130 (23.8%) partners. During labor and childbirth, severe vaginal bleeding was also the most commonly mentioned danger sign by pregnant women (123, 22.5%) and their partners (107, 19.6%). Loss of consciousness or convulsions was the next most commonly mentioned danger sign and was cited by 97 pregnant women (17.8%) and 88 (16.1%) male partners. During the 42-day postpartum period, the most recalled danger sign was fever. This key danger sign was identified by 109 pregnant women (20.0%) and 58 male partners (10.6%). The next most commonly identified danger sign of the postpartum period was severe bleeding. This was mentioned by 68 pregnant women (12.5%) and 54 (9.9%) male partners. Regarding neonatal danger signs, the most recalled danger sign was fever. This was identified by 151 (27.7%) pregnant women and 99 (18.1%) of male partners. The second most common danger sign was convulsions. This was cited by 70 (12.8%) pregnant women and 52 (9.5%) partners (Table 4).

**Table 4:** Sex differences on recalled danger signs.

Variable	Female n_1_ = 546		Male n_2_ = 546		Total n_1_+n_2_ = 1092		p-value
	Yes(n_1,(%))_	No (n_1,(%))_	Yes(n_2,(%)_	No(n_2,(%))_	Yesn_1_+n_2_	Non_1_+n_2_(%	
**During pregnancy**							
Vaginal bleeding	192(35.2)	354(64.8)	130(23.8)	416(76.2)	322(29.5)	770(70.5)	0.000
Swelling of the face and hands	29(5.3)	517(94.7)	25(4.6)	521(95.4)	54(4.9)	1038(95.1)	0.577
Convulsions or unconsciousness	98(17.9)	448(82.1)	51(9.3)	495(90.7)	149(13.6)	943(86.4)	0.000
Severe headache	66(12.1)	480(87.9)	34(6.2)	512(93.8)	100(9.2)	992(90.8)	0.001
Severe abdominal pain	64(11.7)	482(88.3)	48(8.8)	498(91.2)	112(10.3)	980(89.7)	0.111
High fever	96(17.6)	450(82.4)	103(18.9)	443(81.1)	199(18.2)	893(81.8)	0.583
No fetal movement	31(5.7)	515(94.3)	37(6.8)	509(93.2)	68(6.2)	1024(93.8)	0.452
**Labor and childbirth**							
Severe bleeding	123(22.5)	423(77.5)	107(19.6)	439(80.4)	230(21.1)	862(78.9)	0.235
Retained placenta	31(5.7)	515(94.3)	7(1.3)	539(98.7)	38(3.5)	1054(96.5)	0.000
Labor lasting >12hours	36(6.6)	510(93.4)	17(3.1)	529(96.9)	53(4.9)	1039(95.1)	0.007
Loss of consciousness or convulsion	97(17.8)	449(82.2)	88(16.1)	458(83.9)	185(16.9)	907(83.1)	0.468
Severe headache	16(2.9)	530(97.1)	24(4.4)	522(95.6)	40(3.7)	1052(96.3)	0.197
High fever	57(10.4)	489(89.6)	41(7.5)	505(92.5)	98(9.0)	994(91.0)	0.090
Mal-presentation	21(3.8)	525(96.2)	8(1.5)	538(98.5)	29(2.7)	1063(97.3)	0.014
**42 days post delivery**							
Severe bleeding	68(12.5)	478(87.5)	54(9.9)	492(90.1)	122(11.2)	970(88.8)	0.179
Difficulty in breathing	16(2.9)	530(97.1)	10(1.8)	536(98.2)	26(2.4)	1066(97.6)	0.234
Loss of consciousness or convulsion	51(9.3)	495(90.7)	35(6.4)	511(93.6)	86(7.9)	1006(92.1)	0.072
Malodorous vaginal discharge	44(8.1)	502(91.9)	33(6.0)	513(94.0)	77(7.1)	1015(92.9)	0.194
Severe headache	50(9.2)	496(90.8)	28(5.1)	518(94.9)	78(7.1)	1014(92.9)	0.010
Severe weakness	11(2.0)	535(98.0)	9(1.6)	537(98.4)	20(1.8)	1072(98.2)	0.652
Fever	109(20.0)	436(80.0)	58(10.6)	488(89.4)	167(15.3)	924(84.7)	0.000
**Neonatal period**							
Fever	151(27.7)	395(72.3)	99(18.1)	447(81.9)	250(22.9)	842(77.1)	0.000
Excessive crying	30(5.5)	516(94.5)	31(5.7)	515(94.3)	61(5.6)	1031(94.4)	0.895
Unable to breastfeed	63(11.5)	483(88.5)	46(8.4)	500(91.6)	109(10.0)	983(90.0)	0.086
Jaundice	24(4.4)	522(95.6)	24(4.4)	522(95.6)	48(4.4)	1044(95.6)	1.0
Inability to stool	19(3.5)	527(96.5)	14(2.6)	532(97.4)	33(3.0)	1059(97.0)	0.377
Bleeding in the umbilical cord	28(5.1)	518(94.9)	35(6.4)	511(93.6)	63(5.8)	1029(94.2)	0.364
Bad smell discharge from the umbilical cord	38(7.0)	508(93.0)	15(2.7)	531(97.3)	53(4.9)	1039(95.1)	0.001
Convulsion	70(12.8)	476(87.2)	52(9.5)	494(90.5)	122(11.2)	970(88.8)	0.084

### Knowledge of preparations to be made for childbirth

On recalling the preparations to be made for childbirth, only 40 (7.3%) of pregnant women and 32 (3.9%) of their partners managed to recall at least three. The most recalled preparations for childbirth were saving money and preparing items for childbirth. These activities were cited among 392 (71.8%) of pregnant women and 322 (59.0%) of their partners. Knowing the expected date for childbirth and identifying a health facility where they expect their child to be born was the least recalled component of birth preparedness.

### Sex differences on knowledge of birth preparedness and complication readiness

There was a significant difference on mean scores in all dependent variable tested between pregnant women and their partners (Table 5)

**Table 5:** Sex differences in knowledge about birth preparedness and complication readiness.

Variable	Pregnant women Their partners			
	Mean (SD)	Mean (SD)	t	p value
Knowledge scores on danger	M = 3.17(3.793)	M = 2.31(3.298)	t(1069) = -3.968	0.000
Knowledge scores on antenatal care	M = 1.33(0.777)	M = 1.18(0.864)	t(1078) = -3.934	0.001
Knowledge score on preparations	M = 1.09(1.079)	M = 0.88(0.994)	t(1090) = -3.324	0.001
Knowledge score on BRCR	M = 5.58±4.591	M = 4.37±4.285	t(1085) = -4.525	0.000

### Predictors of knowledge among both pregnant women and their partners

Multiple linear regressions were calculated to predict scores on birth preparedness and complication readiness. Based on socio-demographic characteristics, a significant regression equation was found; pregnant women F(14,531) = 5.942; p<0.001, R^2^ = 13.5% and male partner F(12,533) = 5.817; p<0.001, R^2^ = 11.6%. Predictors of knowledge among pregnant women were the age of respondent (β = 0.236; p< 0.01), ethnic group [Mambwe (β = 0.184; p<0.001)], characteristic of health facility [health center (β = 0.103;p<0.05)], having access to media (β = -0.115; p<0.01), having ever heard about birth preparedness (β = 0.171; p<0.001) and having ever had a preterm delivery (β = 0.088; p<0.05) (Table 6). Predictors of knowledge among male respondents were age at marriage (β = -0.103; p<0.05), education level [Primary level (β = 0.157; p<0.001), Secondary level (β = 0.131;p<0.01) ], having access to media (β = -0.168; p<0.0001) and having ever heard about birth preparedness (β = 0.169;p< 0.001) (Table 7).

**Table 6:** Predictors of knowledge after adjusting for the confounders among pregnant women (N = 546).

				p	95.0% CI		Collinearity Statistics	
	B	Beta	t	Value	Lower Bound	Upper Bound	Tolerance	VIF
(Constant)	1.137		0.682		-2.137	4.41		
Age of the respondent in years	0.098	0.146	3.334	***	0.04	0.156	0.85	1.176
Age at marriage in years	-0.013	-0.006	-0.137		-0.197	0.171	0.827	1.21
Primary School	0.378	0.041	0.966		-0.391	1.147	0.902	1.109
Secondary school	2.203	0.083	1.917		-0.055	4.461	0.86	1.163
Mambwe	2.043	0.184	4.075	***	1.058	3.028	0.795	1.259
Others	-0.117	-0.01	-0.227		-1.133	0.898	0.832	1.202
Access to media	-1.06	-0.115	-2.789	**	-1.807	-0.313	0.954	1.049
Health centers	1.252	0.103	2.27	*	0.168	2.336	0.79	1.266
Walking distance 1-5km	-0.055	-0.006	-0.137		-0.845	0.735	0.865	1.156
Walking distance more than 5km	-0.569	-0.037	-0.78		-2.002	0.864	0.711	1.407
Covered with NIHF	-0.062	-0.006	-0.152		-0.87	0.746	0.924	1.082
Ever heard about birth preparedness	2.077	0.171	4.061	***	1.072	3.082	0.919	1.089
Have a preterm delivery	1.8	0.088	2.077	*	0.097	3.504	0.906	1.104
Pre C Section	0.816	0.027	0.64		-1.687	3.319	0.907	1.102

Here *, ** and *** indicate p<0.05, p<0.01 and p<0.001 respectively

**Table 7:** Predictors of knowledge after adjusting for the confounders among male partners (N = 546).

				p	95% CI		Collinearity Statistics	
	B	Beta	t	Value	Lower Bound	Upper Bound	Tolerance	VIF
(Constant)	3.38		2.903	**	1.093	5.667		
Age of the respondent in years	0.045	0.082	1.83		-0.003	0.094	0.836	1.196
Age at marriage in years	-0.113	-0.103	-2.326	*	-0.208	-0.018	0.839	1.191
Primary School	1.405	0.157	3.487	***	0.614	2.196	0.82	1.22
Secondary school	2.2	0.131	2.868	**	0.693	3.708	0.798	1.254
Mambwe	0.876	0.084	1.95		-0.007	1.758	0.89	1.124
Others	0.435	0.032	0.763		-0.685	1.556	0.942	1.061
Own a radio	-1.453	-0.168	-4.023	***	-2.163	-0.744	0.948	1.055
Health centers	-0.502	-0.044	-1.005		-1.482	0.479	0.856	1.168
Walking distance 1–5	0.073	0.008	0.192		-0.671	0.817	0.867	1.153
Walking distance more than 5	0.768	0.054	1.183		-0.508	2.044	0.796	1.257
Covered with NIHF	0.321	0.035	0.813		-0.454	1.095	0.913	1.095
Ever heard about birth preparedness	1.859	0.169	4.068	***	0.961	2.757	0.959	1.043

Here *, ** and *** indicate p<0.05, p<0.01 and p<0.001 respectively

## Discussion

Birth preparedness and complication readiness knowledge is derived from a combination of knowledge about obstetric and neonatal danger signs and core knowledge about antenatal care and childbirth preparations. Knowledge about danger signs of obstetric complications among expectant parents is the key factor that influences timely access to care. Given the impact that BPCR has been shown to have on maternal and neonatal outcomes [17], this study was undertaken to assess BPCR among expecting couples in this rural setting.

Although the majority of study participants stated that they had heard about birth preparedness, the overwhelming majority had inadequate knowledge about obstetric and neonatal danger signs. Among pregnant women, the mean knowledge score on all key danger signs was 3.17±3.793 and among male partners it was 2.31±3.298. The differences in mean score between pregnant women and their partners were statistically significant, meaning that the knowledge about danger signs among pregnant women was significantly higher than that of their partners. A primary reason for this gender gap is inadequate male involvement in maternal health services in Tanzania[17].

Although the majority of study participants stated that they had heard about birth preparedness, they had insufficient knowledge of obstetric danger signs. The mean knowledge score on danger signs among pregnant women was 3.17±3.793, and that of their partners was 2.31±3.298. The differences in mean scores between pregnant women and their partners were statistically significant, meaning that the knowledge about danger signs among pregnant women was higher than that of their partners. This can be explained to be due to limited male involvement in maternal health services [11,17,27].

Danger signs during pregnancy were vaginal bleeding, swelling of face and hands, convulsion severe headache, severe abdominal pain and decreased fetal movement. Vaginal bleeding was the most recalled danger sign among both pregnant women (35.2%) and their partners (23.8%). Vaginal bleeding was also reported to be the most recalled danger sign during pregnancy among male respondents in a previous study in Tanzania [17] although with the proportion of 10.1%. Similarly, vaginal bleeding was the most recalled danger sign among female respondents in other studies [28–30]. Vaginal bleeding is an outwardly visible danger sign of pregnancy compared to other symptoms such as reduced fetal movement. The majority of male and female respondents in this study did not mention swelling of face and hands, convulsion, severe headache, severe abdominal pain or cessation of fetal movement as danger signs during pregnancy. The inability to recognize obstetric danger signs is a key barrier to access obstetric services during obstetric emergencies [31].

Danger signs during labor and childbirth were severe bleeding, convulsion, retained placenta, labor more than twelve hours, severe headache, high fever, and malpresentation. Once again, the most recalled danger sign among both pregnant women (22.5%) and their partners (19.6%) was severe vaginal bleeding. Severe vaginal bleeding was also the most recalled danger sign (22.7%) during labor and childbirth in a previous study conducted among male respondents in rural Tanzania[17]. Knowledge about vaginal bleeding at the time of birth is likely linked to personal experience as globally, postpartum hemorrhage (PPH) is the leading cause of maternal death [2]

The assessed danger signs during 42-days post delivery were severe vaginal bleeding, difficulty in breathing, convulsion, malodorous discharge, severe headache, severe weakness and fever. Knowledge was universally low. The most recalled danger sign among both pregnant women and their partners was fever followed by severe vaginal bleeding. This is contrary to other studies where severe vaginal bleeding was the most recalled danger sign [29]. On a global scale, most maternal mortality occurs during childbirth and the early postpartum period is due to PPH and eclampsia, with sepsis also a common occurrence[15]. Failure of the majority of study participants to identify key danger signs in the postpartum period demonstrates a worrisome gap in basic life-saving knowledge that impacts maternal survival. When it comes to dangers signs during neonatal period, the most recalled danger sign among both pregnant women and their partners were fever (22.9%). Again, both the rate of knowledge and specific content was low, with a worrisome impact on neonatal survival.

When it comes to recalled danger signs, the vast majority of male partners could not recall any danger signs during pregnancy (58.8%), labor (67.8%), 42 days postpartum (76.6%) and the neonatal period (69.8%). This knowledge gap severely impacts timely access to health facility for skilled assistance in case of complications [32]. Similarly, knowledge among pregnant women was minimal with only 14.8%, 6%, 8% and 10.4% of pregnant women able recall at least three danger signs during pregnancy, labor, 42 days post delivery and the neonatal period respectively. This finding corresponds with a study conducted in Mpwapwa District in Dodoma Region, Tanzania where a similarly low proportion of women managed to recall at least three danger signs [33].

Saving money and preparing items to be used during childbirth were the most recalled childbirth preparation activities among both pregnant women and their partners. However, knowing the expected date of delivery and identifying location for childbirth was the least recalled component of birth preparedness. Less than 10% of both pregnant women and their partners knew to identify female relative to escort the woman during labor to a health facility and to identify a relative to take care of siblings as part of birth preparedness.

Predictors of knowledge among pregnant women in this cohort were the age of a woman, ethnic group, level of health facility, ever having had preterm delivery and having ever heard about birth preparedness. Pregnant women with a previous history of preterm birth were more knowledgeable in our study cohort. This finding corresponds with a study conducted in Nigeria by Ekabua et al.(34).

The BPCR knowledge also increased as a woman advanced in age in our study population. This could be due to the impact of a woman’s personal experiences with reproductive health issues both in her own life and among women in her family and community. A study conducted in Uganda found that younger women were more knowledgeable than older women[31]. The difference could be due to differences in the study population. The study in Uganda included both rural and urban population with multiple sources of maternal health education while our study included only rural communities with limited access to outside health facilities maternal health information sources. Among men included in our study, increasing age did not contribute to knowledge about birth preparedness and complication readiness. This difference again highlights the lack of male involvement in pregnancy and childbirth in rural Rukwa Region. Educational status predicted knowledge about birth preparedness and complication readiness among partners but did not significantly influence knowledge among pregnant women, which is contrary to previous study done in Rural Tanzania and Uganda where levels of education of a woman influenced their level of knowledge [32,33,34]. Previous experience of maternal complications such as preterm birth was found to be one of the predictors of knowledge. This finding is in line with prior studies[34].

Educational status predicted knowledge of birth preparedness and complication readiness among partners but did not significantly influence knowledge among pregnant women. Overall, the education level of this cohort of women was quite low, with few women continuing education beyond primary school. This is contrary to other studies where levels of education of a woman influenced their level of knowledge [32,34].

### Conclusion

This study highlights the low levels of BPCR knowledge among a rural population in the Southern Highlands of Tanzania. Furthermore, it revealed gender differences in the levels of knowledge about birth preparedness and complication readiness and the predictors of that knowledge among both women and men. The predictors of knowledge among women were age, having ever heard of birth preparedness, being Mambwe in ethnic origin, living nearby a health center (rather than dispensary) and having ever had a prior preterm delivery. Predictors of knowledge among men were having heard of birth preparedness and educational status. The mean knowledge scores among both men and women were unacceptably low. These findings reveal a need for innovative community-based educational strategies to increase the levels of knowledge about birth preparedness and complication readiness among both men and women.

## Supporting information

S1 File Knowledge on BPCR. (SAV)

## Abbreviations

BPRC: Birth preparedness and Complication Readiness
